# Prevalence of Helminths in Small Ruminant Farms and Evaluation of Control Practices Used to Counter Anthelmintic Resistance in Southern Italy

**DOI:** 10.3390/pathogens13060493

**Published:** 2024-06-09

**Authors:** Fabio Castagna, Roberto Bava, Marta Gagliardi, Simone Russo, Giusi Poerio, Stefano Ruga, Carmine Lupia, Giuseppe Cringoli, Antonio Bosco, Laura Rinaldi, Ernesto Palma, Domenico Britti, Vincenzo Musella

**Affiliations:** 1 Department of Health Sciences, University of Catanzaro Magna Græcia, 88100 Catanzaro, Italy; castagnafabio@yahoo.it (F.C.); roberto.bava@unicz.it (R.B.); rugast1@gmail.com (S.R.); britti@unicz.it (D.B.); musella@unicz.it (V.M.); 2 Mediterranean Ethnobotanical Conservatory, Sersale (CZ), 88054 Catanzaro, Italy; studiolupiacarmine@libero.it; 3 Interdepartmental Center Veterinary Service for Human and Animal Health (CISVet-SUA), University of Catanzaro Magna Graecia, 88100 Catanzaro, Italy; martag956@gmail.com (M.G.); simoneru86@libero.it (S.R.); 4 ATS Val Padana, Via dei Toscani, 46100 Mantova, Italy; giusipoerio@gmail.com; 5 Department of Veterinary Medicine and Animal Production, University of Naples Federico II, CREMoPar, 80100 Napoli, Italy; cringoli@unina.it (G.C.); antonio.bosco@unina.it (A.B.); lrinaldi@unina.it (L.R.); 6 Center for Pharmacological Research, Food Safety and High Tech and Health (IRC-FSH), University of Catanzaro Magna Græcia, 88100 Catanzaro, Italy

**Keywords:** helminth control, small ruminants, anthelmintic treatments, anthelmintic resistance, health surveillance, animal welfare and health

## Abstract

Anthelmintic resistance in small ruminants is a serious worldwide problem. To reduce their spread, it is essential to know the prevalence of helminths on farms and the control practices adopted. As these studies in the Calabria region of southern Italy are fragmentary and outdated, a study on the prevalence of helminths in small ruminant holdings in this area has been conducted. The measures implemented to control helminths were also evaluated through questionnaires administered to farmers. In particular, on 90 farms (45 sheep and 45 goats), 1800 faecal samples from 900 sheep and 900 goats were collected in the spring. Using the FLOTAC dual technique, parasitological examinations demonstrated the presence of gastrointestinal nematodes in 100% of sheep and goat farms, followed by *Nematodirus* spp. (84.44% sheep and 48.89% goats), *Moniezia* spp. (73.33% sheep and 35.56% goats), *Trichuris ovis* (48.89% sheep and 42.22% goats), lungworms (28.89% sheep and 42.22% goats), *Strongyloides papillosus* (40% sheep and 26.67% goats), *Dicrocoelium dendriticum* (13.33% sheep and 26.67% goats), *Calicophoron daubneyi* (6.67% sheep and 31.11% goats), *Fasciola hepatica* (6.67% sheep and 4.44% goats), and *Skrjabinema ovis* (4.44% sheep and goats). The questionnaires showed that 82% and 85% of the farmers had applied pasture rotation, and that 93.3% and 86.6% had used anthelmintics in the previous year for sheep and goats, respectively. Only 24.4% of sheep farmers and 11.3% of goat farmers had carried out parasitological tests prior to treatments. The most used classes of anthelmintics were macrocyclic lactones and benzimidazoles, and only in 21.6% and 15.6%, for sheep and goats, respectively, was drug rotation carried out. These results denote that helminths represent a health problem for small ruminants and highlight a lack of knowledge of parasite control strategies among farmers. In these conditions, anthelmintic resistance phenomena could develop over time. Therefore, it is necessary to implement all possible strategies for the control of helminths, and to prevent the spread of anthelmintic resistance phenomena on farms in southern Italy.

## 1. Introduction

In Europe, dairy sheep and goat populations are mainly distributed in the countries of the Mediterranean basin. Greece and Italy are among the countries with the largest dairy sheep flocks, while Greece has the largest concentration of goat flocks [1].

The Italian livestock population, referring to sheep and goat species, consists of 6,996,536 million heads, of which 5,981,659 million are sheep and 1,014,877 million are goats (updated as of 31 March 2023) [2]. Most of the Italian small ruminant farms are located in the central and southern regions of the peninsula, as well as on the major islands. The Calabria region of southern Italy has a strong agro-pastoral economy, and raises a considerable number of sheep and goats. Specifically, small ruminant livestock breeding is widespread in that region (10,113 farms), for a total of 187,445 sheep and 109,425 goats (31 March 2023). These data were provided by the National Data Bank (BDN) of the Livestock Registry—CSN of the “G. Caporale” Institute in Teramo, Italy [2]. A careful analysis of the data shows that the region is ranked fifth nationally for the number of sheep, second for goats, fifth for the quantity of goat farms, and fourth for the quantity of sheep and mixed breeders.

Sheep and goat farms are mainly found in less developed rural and mountainous areas, where production systems are closely linked to local traditions and have extensive farming systems. These farming systems are essential for maintaining rural areas [3] and are an important source of family income [1]. This is also true for the Calabria region, where dairy sheep and goat farming is an important economic resource, especially in mountainous and hilly areas, with significant income for the weak agri-food sector. In particular, milk processing produces important traditional dairy products with the Protected Designation of Origin (D.O.P.) mark and many cheeses and dairy products Traditional Italian Food Products (P.A.T.). The Calabria Region has 268 P.A.T. and, among them, numerous dairy specialties (cheeses and dairy products) from goats and sheep [4]. 

In addition to the milk production, sheep and goat farming plays an important role in the conservation of mountain landscapes, especially at high altitudes and on steep slopes, and is closely linked to agritourism activities [5]. Furthermore, the grazing system has been an important component of the Mediterranean ecosystem for centuries, and a valuable tool for environment management and conservation [6,7,8].

More than 91% of the Calabrian territory consists of mountains (41.8%) and hills (49.3%). Plains (8.9%) are limited to coastal areas (Tyrrhenian and Ionian areas), and most farms, mainly extensive, are distributed in marginal areas. The pasture-based livestock system brings benefits to both the environment and mountain populations, with unquestionable advantages for dairy production related to pasture milk. In these farming conditions, the milk is richer than in stable farming for its higher content of healthful compounds and its hedonistic characteristics [9]. However, all grazing animals are more susceptible to helminth infections, and any future intensification of livestock farming will increase the risk of helminth infections and diseases [10]. These represent a serious threat to the health of small ruminants globally, with important impacts on productivity and welfare and increasing veterinary costs [11].

In addition, high levels of infection lead to increased energy and protein requirements, resulting in lower nutrient availability for production [12,13]. These infections are of economic importance not only in mountain areas but worldwide and result in deaths, reduced weight gain, and organs being discarded at slaughter [14,15,16,17]. To these must be added the appreciable losses in milk production, both in terms of quantity and quality [18,19,20,21,22,23]. They may also act as vehicles for the transmission of other infectious agents, serving as a gateway for subsequent infections [24]. Parasitic infections continue to pose a serious challenge to the health, welfare, productivity, and reproduction of grazing ruminants worldwide [10]. Therefore, knowledge of the presence and distribution of helminthic infections is essential for planning effective parasite control programs [25].

Recent studies indicate that the annual cost of helminth infections in dairy goats and sheep is about 25 million euros. These expenses are mainly attributable to production losses and treatment costs. Specifically, the study estimates losses of about 22.5 million and more than 2.5 million for sheep and dairy goats, respectively [26]. 

Currently, helminth control is mainly based on the use of synthetic anthelmintics and well-defined control strategies, which contribute significantly to maintaining good health and welfare and improving animal productivity on extensive livestock farms [27]. The latter should be based on diagnosis, proper use of anthelmintics, pasture management, refugia strategies, and the possible use of bioactive botanical species. In the absence of globally usable vaccines, it is imperative to follow the above-mentioned practices [28]. Nevertheless, the indiscriminate use of drugs and improper control practices over the years have led to the development of widespread drug resistance phenomena to one or more classes of anthelmintics globally [29]. 

In recent decades, anthelmintic resistance (AR) phenomena have spread like wildfire in Europe, also affecting Italy [30]. In Italy, initially, these phenomena mainly affected the Central and Northern regions [31,32,33,34]. Recently, AR phenomena have been observed in the territories of southern Italy, where this problem had never been observed [35]. 

Climate change issues must be added to this. Recently, climate change, land use, and farm management have challenged the established control programs [36]. For example, in high-temperature regions, prolonged grazing seasons under warmer autumn–spring conditions present new opportunities for parasite transmission [37], while hot and dry summers can drive biphasic peaks in infective stage development [29]. Therefore, helminth control programs need to be reevaluated and adapted to maintain their efficacy [36].

Southern Italy, particularly the Calabria region, has an important livestock vocation, still based on extensive farming of small ruminants, with excellent agrifood production. This valuable region should be better protected and enhanced, including through the planning of parasite infection control programs, which would significantly affect livestock production, animal welfare, and health. However, to plan effective parasite control plans, also aimed at reducing the spread of AR in southern Italy, it is necessary to know the parasite prevalence in small ruminant herds and to evaluate the practices put in place to control them. Only then can appropriate control systems be developed. 

Epidemiological data on the distribution of gastrointestinal nematodes (GINs), tapeworms, liver worms, and ruminal worms in Italian small ruminants are outdated and fragmentary [25]. Therefore, since studies conducted in this area are scarce and the latest research on the prevalence of parasites dates to the 1990s, a study was conducted to acquire recent data on the prevalence of helminths in pasture-raised sheep and goats in the Calabria region of southern Italy. In addition, a questionnaire was conducted to determine and evaluate current parasite control practices on small ruminant farms.

## 2. Materials and Methods

### 2.1. Study Area

The study was conducted in spring, between April and May, on dairy farms in southern Italy dedicated to extensive breeding, with sheep and goats reared on lowland and hilly pastures. The geographical area of study was the Ionian side (Gulf of Squillace) in the Province of Catanzaro (Calabria region).

The farms were selected after careful analysis of the data extracted from the National Data Bank (BDN) [2]. Out of a total of 1537 small ruminant farms present in the Province of Catanzaro (878 sheep farms and 669 goat farms), 330 registered sheep farms and 222 goat farms, present on the Ionian coast of Catanzaro, at an altitude between 0 and 600 m above sea level (average altitude of 398 m above sea level), were identified.

Subsequently, those with at least 50 animals of each species were selected, resulting in a total of 120 sheep farms and 141 goat farms. Of these, based on the farmers’ availability, in relation to the fact that there had to be at least 50 animals on the farm and that the animals had not been subjected to anthelmintic treatments for at least six months, 90 small ruminant farms were selected. These consisted of 45 sheep and 45 goat farms, with a total of 900 sheep and 900 goats raised on pasture.

### 2.2. Survey

Through careful farm screening, the preventive measures and practices implemented for endoparasite control were examined. For this purpose, a questionnaire was administered to each farm owner. All farmers were asked about their farm management practices, i.e., farming system, number of animals, breeds raised, pasture size, and helminth control practices.

It was also asked whether parasitological tests were carried out, the types of tests, the products and dosages of anthelmintic drugs possibly used to control endoparasites, and the timing, frequency of treatments, and rotation of the anthelmintic drugs.

The questionnaires were filled out by interviewing the farmers at the time of the farm visit for sample collection.

### 2.3. Sampling of Faeces

The search for parasite elements (eggs and larvae) required the collection of coprological material in the field, for a total of 1800 individual faecal samples, of which 900 were from the sheep species and 900 from the goat species. Individual faecal sampling was conducted in the morning, on average at 6:00 a.m., before milking.

In each sheep and goat farm, individual faecal samples (10–20 g) were taken at random directly from the rectal ampulla to minimize the risk of environmental contamination. Sampling was carried out on 15 adult females and 5 female lambs per species [38,39]. The latter consist of replacement lambs, with an average age of 6 months, intended to replace dairy ewes at the end of their career or slaughtered due to health or reproductive problems.

The samples from each farm, accompanied by a farm card and identification code, were sent at refrigeration temperature to the Interdepartmental Service Center (CISVetSUA)—Veterinary Services for Human and Animal Health” of the University “Magna Græcia” of Catanzaro, where all the parasitological research was carried out.

### 2.4. Laboratory Analysis

In the CIS-VetSUA laboratories, the faecal samples from each farm were analysed as a pool of five individual samples. In particular, 4 pools (each composed of equal parts of 5 individual samples) were created: a pool related to the replacement lambs and 3 pools related to the adult sheep [38].

Faecal egg count (FEC) was determined by the FLOTAC dual technique with a sensitivity of 2 eggs per gram (EPG) of faeces using two flotation solutions (FS) with different specific gravity (s.g.) [40]. In particular, a sodium chloride-based flotation solution (FS2—NaCl, s.g. 1200) was used to detect the eggs of the gastrointestinal nematodes *Nematodirus*,* Strongyloides* spp., *Skrjabinema*, and* Moniezia* spp., and a zinc sulphate-based flotation solution (FS7—ZnSO_4_-7H_2_O, s.g. 1350) was used in order to detect *Trichuris* spp., Trematoda eggs (*Dicrocoelium dendriticum*,* Calichophoron daubneyi*, and *Fasciola hepatica*), and lungworm larvae.

The choice of the FLOTAC technique for the study fell on the grounds that it is a sensitive and accurate multivariate microscopic technique for the examination of faecal samples, particularly suitable for the detection and counting of parasitic elements in small ruminants [40], including lungworms. In addition, for further identification of GIN genera, eggs from the pooled faeces were cultured to obtain third-stage larvae (L3), which were recovered from the coprocultures by baermannisation [41] and identified according to the keys by Van Wyk et al. [42]. When a coprocolture had 100 or fewer L3, all were identified; when more than 100 L3 were present, only the first 100 examined were identified. The percentage of larval type was calculated based on the counted L3 when fewer than 100 L3 were isolated from a sample.

For greater diagnostic accuracy, a pooled sample of each farm was used to detect the eggs of liver flukes, applying the sedimentation test using 5–10 g of faeces, and the larvae of lungworms, applying the Baermann test using approximately 4 g of faeces [41].

### 2.5. Statistical Analysis

Data were analysed by GraphPad Prism 9 (GraphPad Sofware, Inc., La Jolla, CA, USA) using the Chi-square test to compare the difference in the percentage of GINs and lungworms between sheep and goats. After the log transformation of faecal egg count, the mean FEC was analysed using ANOVA. Normality was tested using the Shapiro–Wilk test. Bartlett’s test was used to assess the homogeneity of variance. A *p*-value less than 0.05 was considered significant at a 95% confidence level.

## 3. Results

### 3.1. Survey

All the farms examined specialized in milk production and practiced a semi-extensive farming system, with animals grazing between March/April and October/November. In the colder months, the animals were housed in barns. A total of 82% of the sheep farms and 85% of the goat farms applied pasture rotation; the remaining farms kept their flocks on the same pastures.

In sheep breeding, the predominant breeds were the Sarda and Lacaune, followed by crosses with local breeds and the Comisana, while in goat breeding, the predominant breeds were local breeds, Nicastrese and Rustica di Calabria, in particular, followed by crossbreeds, the Camosciata delle Alpi and Maltese.

In the previous year, 93.3 % and 86.6% had used anthelmintics, respectively, for sheep and goats. Unfortunately, only 24.4% of sheep farmers and 11.3% of goat farms performed parasitological tests before treatment. The most widely used classes of anthelmintics were macrocyclic lactones and benzimidazoles. Only in 21.6% and 15.6%, respectively, for sheep and goats was drug rotation carried out. Anthelmintic efficacy has never been evaluated on any farm.

The results relating to the control strategies adopted for helminths, the parasitological examinations performed, the type of examinations, the anthelmintic drugs used for antiparasitic treatments, the timing, and the frequency of treatments are shown in Table 1 and Table 2.

### 3.2. Parasitological Analysis

The results of the parasitological analyses with the number of positive farms (sheep, goats), species and/or group of helminths, prevalence (C.I. 95%), mean intensity expressed in eggs or larvae per gram (EPG or LPG) of faeces, and the positivity to cestodes are shown in Table 3 and Table 4.

The FEC of *Nematodirus* spp., *S. ovis*, and *F. hepatica* showed no significant differences when compared between sheep and goats. That of *S. papillosus*, however, showed significantly (*p* > 0.001) higher levels of FEC in sheep than goats. Also, the FEC of *D. dendriticum* was found to be significantly (*p* > 0.001) higher in sheep than in goats. *T. ovis* and *C. daubneyi* FECs were significantly (*p* > 0.001) higher in goats when compared with FECs of these helminths in sheep.

Gastrointestinal nematodes were found on all sheep and goat farms examined in this study, so there was no significant difference in prevalence (*p* > 0.05). Instead, *Nematodirus* spp. prevalence was significantly higher in sheep farms than in goat farms (*p* < 0.05). There was also a higher prevalence of *S. papillosus* in sheep than that found in goat farms (*p* < 0.001). *S. ovis*, *T. ovis*, and *F. hepatica*, on the other hand, showed no significant differences in prevalence between the two animal species (*p* > 0.05).

The same trend was observed for *Moniezia* spp., with an even higher prevalence in sheep than in goats (*p* < 0.001). The prevalences of lungworm, *D. dendriticum*, and *C. daubneyi* were significantly lower (*p* < 0.05 and *p* < 0.001, respectively) in sheep than in goat farms. The prevalence of *Moniezia* spp. found in sheep farms was significantly higher than that obtained in goat farms (*p* < 0.001).

The percentages of GIN genera, detected in sheep and goat farms by coproculture, are shown in Table 5, while the species of lungworms present, detected using the Baermann technique, are reported in Table 6.

## 4. Discussion

The results of this study have highlighted a number of important problems in parasite control on small ruminant farms in southern Italy. The practices used could favour the spread of AR phenomena over time, as well as negatively impact animal health and welfare. Specifically, the data collected on the antiparasitic prophylaxis measures adopted showed that most farmers use anthelmintic drugs for parasite control on their farms, with treatments usually not preceded by parasitological exams and carried out mainly on an annual basis, between February and March. Unfortunately, similar practices have been found in other Italian regions, both in sheep and goat farming [32,43,44].

The most used anthelmintic drugs were ivermectin, albendazole and netobimine, which were rarely rotated. Few farmers treated their animals in the peripartum period, particularly in goat farming. Furthermore, these treatments were imprecise, particularly in goat farms, where most farmers used the same drug dosages as those used for sheep. These results are in line with other studies conducted in Italy [43] and are very widespread in goat farming [45,46], despite the different response to anthelmintic drugs by the two species is well proven in the scientific literature [47,48,49]. In addition, the drug efficacy has never been evaluated in both sheep and goat farms.

The parasitological examinations have revealed the presence of different groups and genera of helminths, both in sheep and goats, with different intensities between the two species. This study is in line with other studies conducted in other regions of southern Italy [39] and confirms that small ruminant gastrointestinal nematodes pose a serious threat to the health of small ruminants, particularly those raised on pasture [10]. The low prevalence of pathogenic gastrointestinal nematodes, such as *Haemonchus* and *Teladorsagia*, is in line with that of other studies conducted in southern Italy in the spring season [22,39,50] and would explain why most infected small ruminants are asymptomatic and have mainly subclinical infections, even with high EPG values. However, some species of *Trichostrongylus*, the predominant genus in this study, and, in particular, *T. colubriformis*, often appear as co-pathogens with other gastrointestinal worms under natural conditions [51] (Kaba et al. 2023), especially in late autumn and winter. The absence of overt clinical symptoms would also explain the underestimation of parasite problems among farmers, who, in most cases, treat their animals only when clinical symptoms are present, without any parasitological diagnosis. This study also highlights that sheep and goats have a different parasite physiognomy, which requires a prophylactic therapeutic approach adapted to the species [47,48,49], and that the less effective anthelmintic treatments in goats could contribute to nematode resistance [43].

It has been described that to improve the health and welfare of small ruminants in this area, stronger control strategies are needed, as the control practices that emerged from the study could not only make drug treatments less effective, or even completely ineffective, but could also promote drug resistance phenomena in the helminth population over time, resulting in significant economic losses. Furthermore, the results emphasize a worrying picture that denotes a lack of knowledge on helminth control strategies and the use of anthelmintics. Helminths, particularly gastrointestinal nematodes, have been shown to represent a serious health problem for small ruminant farms in this area. Sustainable helminth control must be effective and therefore requires in-depth knowledge that addresses the problem at the regional, farm, individual, or paddock level, as well as at the herd and individual animal level [28]. As described in a recent study [25], a national strategy is needed to change the attitudes of sheep and goat farmers and veterinary professionals toward a more sustainable approach to parasitosis on sheep and goat farms.

These findings suggest poor knowledge of helminth control strategies and/or reluctance on the part of farmers to apply the knowledge. To ensure the best state of health and welfare for animals, it is necessary to provide correct training to farmers and operators based on strategies to combat helminths, the importance of parasitological diagnosis, and the correct use of the drugs. These actions are also necessary to avoid the spread of AR.

Unfortunately, this study also shows that very few farmers use natural mixtures from ethnoveterinary knowledge as an alternative treatment or in combination with synthetic drugs. To reduce the development and spread of AR phenomena in small ruminants, there is an urgent need to find alternatives to the massive use of synthetic anthelmintics. The advent of AR means that we can no longer rely on the use of anthelmintics only as the single option for helminth control [35].

Such a picture suggests that green veterinary pharmacology could be the future approach to counteract AR in small ruminants while improving animal welfare and health.

Compared to modern veterinary therapies, the use of plants as anthelmintics has some advantages, such as low cost, lack of side effects, and ease of access. In the past, much information on the antiparasitic properties of plants was passed down orally from generation to generation and lacked scientific validity [52].

In recent years, an increasing number of carefully monitored laboratory and field studies have been conducted to verify and measure the anthelmintic activity of plants. The use of biological methods, also based on the use of compounds of natural origin and bioactive plants with proven anthelmintic efficacy in vivo [53,54,55,56], in combination with other strategies (rational use of anthelmintics, drug rotation, accurate parasitological diagnosis, identification of the most favourable period for treatments, etc.), could play a valuable role in the effective control of these parasites in an integrated and environmentally friendly way, significantly reducing drug use and the risk of developing AR phenomena [57,58,59]. In contrast to their unrestricted usage in conventional production, anthelmintics are used more moderately in the organic and integrated farming systems, which should lessen the selection pressure on the spread of AR [60,61]. The development of AR must be detected early to ensure the high anthelmintic efficacy of drugs [10]. In addition, the integration of a standardised, accurate, and practical approach to monitoring the efficacy of anthelmintics is needed to compile an accurate picture of the state of AR worldwide [35].

Therefore, passive surveillance, based on in vitro tests, and active surveillance, based on the use of in vivo tests, are strongly recommended to control the spread of resistance to anthelmintic drugs in small ruminant farms in southern Italy. Likewise, further research is needed to develop alternative approaches to minimise the use of anthelmintics. Furthermore, it would be interesting to extend these studies to the autumn season to assess the seasonality of helminth infections. This would address a limitation of this study, namely, that the monitoring period fell only in the spring.

## 5. Conclusions

In conclusion, diagnosis, proper therapy based on the correct use of drugs, and correct control strategies, as well as local knowledge of the spread of endoparasites, the practices implemented to control them, and passive and active monitoring are the prerequisites for managing parasitic diseases and slowing the spread of AR phenomena in sheep and goat farms in southern Italy.

All this can only be achieved through the proper and continuous training of all professionals and operators in the field. Such an approach will make them more responsible and aware of this very important issue and the practices that must be put in place to counter AR in small ruminant farms.

## Figures and Tables

**Table 1:** Anthelmintic practices used for helminth control in sheep farms.

Worm-Control Factor in Sheep Farms (N Sheep Farms = 45)	N	%
Treatments frequency per year		
None	3	6.67
Only once	22	48.89
Once or twice	9	20
Twice	11	24.44
Pre-treatment parasitological analysis		
None	34	75.56
Once	11	24.44
Anthelmintic classes used		
Macrocyclic lactones	18	40.00
Benzimidazoles	11	24.44
Macrocyclic lactones and benzimidazoles	9	20.00
Natural mixture	7	15.67

**Table 2:** Anthelmintic practices used for helminth control in goat farms.

Worm-Control Factor in Goat Farms (N. Goat Farms = 45)	N	%
Treatment frequency per year		
None	6	13.33
Only once	30	66.67
Once or twice	6	13.33
Twice	3	6.67
Pre-treatment parasitological analysis		
None	40	88.89
Once	5	11.31
Anthelmintic classes used		
Macrocyclic lactones	21	46.67
Benzimidazoles	10	22.22
Macrocyclic lactones and benzimidazoles	14	26.67

**Table 3:** Results of parasitological analyses in sheep farms, parasite intensity expressed as eggs per gram (EPG) of faeces or larvae per gram (LPG) of faeces, standard deviation (SD) and *p*-value.

Parasites	Parasitic Intensity EPG or LPG (SD) (Minimum–Maximum)		*p*-Value
	Sheep	Goat	
Gastrointestinal nematodes	40 (108) (32–3942)	76 (321) (39–4386)	>0.05
*Nematodirus* spp.	12 (17.79) (1–94)	5 (5.83) (1–15)	>0.05
*Strongyloides papillosus*	5 (9.05) (2–72)	2 (3.16) (1–12)	<0.001
*Skrjabinema ovis*	1 (0.40) (2–3)	1 (0.26) (1–2)	>0.05
Lungworm	14 ^(^*^)^ (31.09) (2–155)	19 ^(^*^)^ (65.02) (11–350)	>0.05
*Trichuris ovis*	8 (10.99) (1–136)	23 (17.66) (1–165)	<0.001
*Dicrocoelium dendriticum*	1 (19.90) (9–300)	4 (5.83) (2–17)	<0.001
*Calichophoron daubneyi*	2 (6.43) (12–44)	61 (33.03) (3–324)	<0.001
*Fasciola hepatica*	1 (1.25) (1–18)	1 (1.25) (2–18)	>0.05
*Moniezia* spp.	+	+	

^(^*^)^ values expressed in LPG.

**Table 4:** Results of parasitological studies in sheep and goat farms, including positive farms, parasite prevalences % (C.I. 95%) and *p*-value.

Parasites	Positive Farms		Prevalence % (C.I. 95%)		*p*-Value
	Sheep	Goat	Sheep	Goat	
Gastrointestinal nematodes	45	45	100	100	>0.05
*Nematodirus* spp.	38	22	84.44 (52.79–92.90)	48.89 (33.83–63.02)	<0.05
*Strongyloides papillosus*	18	12	40 (26.06–55.63)	26.67 (16.92–41.25)	<0.001
*Skrjabinema ovis*	2	2	4.44 (1.16–19.05)	4.44 (1.06–21.51)	>0.05
Lungworm	13	19	28.89 (16.84–44.52)	42.22 (23.91–59.46)	<0.001
*Trichuris ovis*	22	19	48.89 (33.94–64.02)	42.22 (26.91–52.46)	>0.05
*Dicrocoelium dendriticum*	6	12	13.33 (5.54–27.49)	26.67 (15.22–42.25)	<0.05
*Calichophoron daubneyi*	3	14	6.67 (1.74–19.31)	31.11 (18.80–45.36)	<0.001
*Fasciola hepatica*	3	2	6.67 (1.74–19.31)	4.44 (1.16–26.51)	>0.05
*Moniezia* spp.	33	16	73.33 (57.79–84.90)	35.56 (16.64–51.20)	<0.001

**Table 5:** Results of GIN coprocultures in sheep and goat farms and Chi-square test.

GIN GENERA	OVERALL %	SHEEP %	GOATS %	X^2^	*p* VALUE
*Haemonchus*	13.5	15	12	0.16	0.685
*Oesophagostomum/Chabertia*	27	28	26	0.37	0.847
*Trichostrongylus*	46	45	47	0.02	0.882
*Teladorsagia*	13.5	12	15	0.16	0.685

**Table 6:** Percentages of lungworm species detected by baermannisation in sheep and goat farms and chi-square test.

LUNGWORMS SPECIES	OVERALL %	SHEEP %	GOATS %	X^2^	*p* VALUE
*Muellerius capillaris*	50.5	45.0	56.0	0.59	0.439
*Cystocaulus ocreatus*	29.0	35.0	23.0	1.25	0.262
*Dictyocaulus filaria*	20.5	20.0	21.0	0.01	0.912

## Data Availability

The data are kept at University Magna Græcia of Catanzaro and are available upon request.
